# Extracellular secretion of a cutinase with polyester-degrading potential by *E. coli* using a novel signal peptide from *Amycolatopsis mediterranei*

**DOI:** 10.1007/s11274-022-03246-z

**Published:** 2022-02-23

**Authors:** Yeqi Tan, Gary T. Henehan, Gemma K. Kinsella, Barry J. Ryan

**Affiliations:** School of Food Science and Environmental Health, Technological University Dublin, Grangegorman, Dublin 7, D07 ADY7 Ireland

**Keywords:** *Actinomycetes*, *Amycolatopsis*, *E. coli*, Cutinase, Signal peptide, Polyester hydrolysis, Extracellular

## Abstract

Recent studies in this laboratory showed that an extracellular cutinase from *A. mediterranei* (AmCut) was able to degrade the plastics polycaprolactone and polybutylene succinate. Such plastics can be slow to degrade in soils due to a lack of efficient polyester degrading organisms. AmCut also showed potential for the biocatalytic synthesis of esters by reverse hydrolysis. The gene for AmCut has an upstream leader sequence whose transcript is not present in the purified enzyme. In this study, we show using predictive modelling, that this sequence codes for an N-terminal signal peptide that directs transmembrane expression via the Sec secretion pathway. *E. coli* is a useful host for recombinant enzymes used in biocatalysis due to the ease of genetic manipulation in this organism, which allows tuning of enzymes for specific applications, by mutagenesis. When a truncated GST-tagged AmCut gene (lacking its signal peptide) was expressed in *E. coli*, all cutinase activity was observed in the cytosolic fraction. However, when GST-tagged AmCut was expressed in *E. coli* along with its native signal peptide, cutinase activity was observed in both the periplasmic space and the culture medium. This finding revealed that the native signal peptide of a Gram-positive organism (AmCut) was being recognised by the Gram-negative (*E. coli*) Sec transmembrane transport system. AmCut was transported into *E. coli’*s periplasmic space from where it was released into the culture medium. Surprisingly, the presence of a bulky GST tag at the N-terminus of the signal peptide did not hinder transmembrane targeting. Although the periplasmic targeting was unexpected, it is not unprecedented due to the conservation of the Sec pathway across species. It was more surprising that AmCut was secreted from the periplasmic space into the culture medium. This suggests that extracellular AmCut translocation across the *E. coli* outer membrane may involve non-classical secretion pathways. This tuneable recombinant *E. coli* expressing extracellular AmCut may be useful for degradation of polyester substrates in the environment; this and other applications are discussed.

**Graphical abstract:**

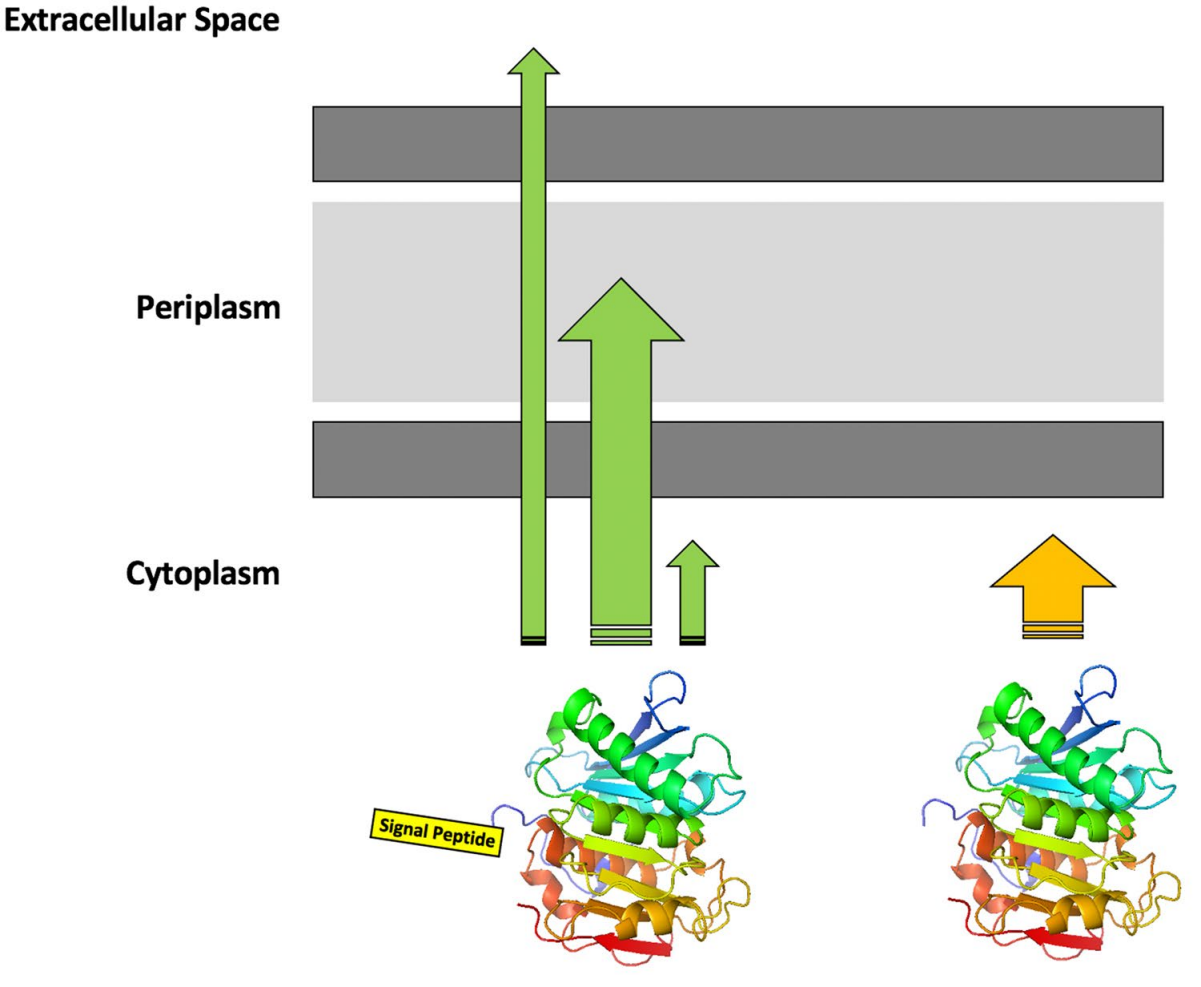

**Supplementary Information:**

The online version contains supplementary material available at 10.1007/s11274-022-03246-z.

## Introduction

Ongoing studies in this laboratory are focussed on the discovery of thermostable, solvent tolerant enzymes with biotechnological applications in areas such as biofuels synthesis, feedstock degradation and green synthesis of industrially useful compounds (e.g. Uhoraningoga et al. 2021; Priyanka et al. 2019; Kumar et al. 2017). In the course of these studies, a novel extracellular cutinase (AmCut) from the Gram-positive organism *Amycolatopsis mediterranei* (AmCut) was identified with potential applications in biotechnology. AmCut was originally identified as a lipase; however, subsequent structural and substrate specificity studies showed it was more appropriately designated as a cutinase (Tan et al. 2021). Lipases and cutinases are closely related acyl hydrolases (Chen et al. 2013). The native AmCut was solvent tolerant, had specificity for medium chain fatty acid esters and was capable of flavour ester synthesis (Dheeman et al. 2011).

In a previous paper, we reported the modelled 3-D structure of AmCut and showed it to have an open, surface exposed, active site and that it was capable of degrading specific polyester plastics such as polycaprolactone and polybutylene succinate but not polylactide or polyethylene terephthalate (Tan et al. 2021). Extracellular expression of this enzyme in *E. coli* offers the intriguing possibility of creating an organism capable of secreting a thermostable, solvent tolerant cutinase for degradation of polyester compounds. Such an organism may be readily tuned to tailor its specificity for specific substrates due to the ease of gene manipulation in *E. coli*.

Cutinases, such as AmCut, are secreted by bacteria to degrade extracellular substrates and to invade host tissues. They are generally robust enzymes since they need to withstand extracellular environments and they have interesting biotechnological properties being able to degrade insoluble triglyceride substrates and polymers such as cutin (see Nyyssölä 2015). The heterologous expression of secreted enzymes such as cutinases in *E. coli* is desirable due to lower production costs and ease of growth (Kleiner-Grote et al. 2018). However, expression of cutinases in *E. coli* has proven problematic and can lead to inclusion body formation, especially when associated with N-terminal signal peptides (Su et al. 2013; Rosano and Ceccarelli 2014). Even lipases, which are closely related to cutinases, can be challenging to produce in recombinant form. For example, Peng et al. (2011) reported inclusion body formation when expressing *Pseudomonas aeruginosa* PSA01 Lipase A in *E. coli* when its signal peptide was present. Production was subsequently improved by co-expression with a chaperone (see Pulido et al. 2020). Membrane-associated lipases can also be difficult to express in *E. coli* due to inclusion body formation, but these expression issues can be circumvented by use of a purification tag and solubility enhancer (such as a maltose-binding protein) as shown by Zhang et al. (2019) for a lipase from *Rhizopus chinensis*, for example.

Heterologous expression of secreted cutinases can also display a tendency to degrade *both* inner and outer *E. coli* membranes and, therefore, become secreted into the culture medium. This can be an advantage in some cases since the secreted enzyme may be readily harvested. Su et al. (2013) showed that a cutinase from *Thermobifida fusca* was secreted into the culture medium when expressed in *E. coli without* a signal peptide. This secreted cutinase was capable of digesting both inner and outer membrane phospholipids and thereby exiting the cell. This digestion did not appear to affect cell growth, although foam generation was problematic when scaled up to larger volumes and cell morphology was altered (Su et al. 2017). The ability of lipases/cutinases to digest *E. coli* cell membranes was exploited to allow for the enhanced leakage of co-expressed proteins. Thus, Su et al. (2017) demonstrated that enhanced extracellular production of proteins was facilitated by co-expression with phospholipase C.

In this present study, the AmCut gene was found to have an upstream leader sequence which coded for a signal peptide following gene sequencing and predictive modelling. The AmCut open reading frame *including* the upstream leader sequence was de novo synthesised commercially, with codon optimisation for heterologous *E. coli* expression and with an incorporated a GST tag to facilitate purification. The GST tag was placed at the N-terminal end of the construct such that the signal peptide leader sequence was located between the AmCut gene and the GST tag. Expression of the AmCut gene constructs with, and without, the signal peptide was explored. Using subcellular distribution studies, we showed that the signal peptide functions to direct the expression of AmCut to the periplasmic space of *E. coli*. From the periplasmic space the cutinase was secreted into the culture medium. Without the signal peptide, expression was only in the cytosol. It was of interest that a signal peptide, designed for transmembrane transport in a Gram-positive organism (*Amycolatopsis*), was capable of functioning with the cellular machinery of a Gram-negative organism, *E. coli*. Additionally, it was capable of directing expression to the periplasmic space, even in the presence of a bulky GST tag. To the best of our knowledge, this is the first extracellular cutinase exhibiting these properties to be expressed in *E. coli* and offers significant potential for biotechnological applications.

## Materials and methods

### Materials

Media components were purchased from Lab M, Bury, UK. Molecular biology materials were from New England Biolabs. All other materials were obtained from Sigma Aldrich and were of analytical grade. The sonicator used for cell lysis was a Q55 Sonicator with standard 1/8” diameter probe (from Qsonica LLC, Newtown, CT, USA).

### Methods

The codon-optimised gene for AmCut cutinase (AmCut; GenBank Accession Number MZ424082) was commercially synthesised by Prozomix (Haltwhistle, United Kingdom), ligated into the pET49b vector and transformed into *E. coli* BL21(DE). The expression system contains a GST tag that allows purification of the recombinant protein by glutathione affinity chromatography. The GST tag was cleaved off by on-column cleavage using PreScission Protease.

### Sequence analysis

Homology searches of DNA and protein databases were performed using the NCBI BLAST server (https://blast.ncbi.nlm.nih.gov/Blast.cgi; Altschul et al. 1990). Multiple sequence alignment was carried out using the Clustal Omega tool. Prediction of the signal peptide was carried out by SignalP-5.0 (http://www.cbs.dtu.dk/services/SignalP/).

### Preparation of *E. coli* expression culture

A 5 ml aliquot of MDG media containing 50 µg/ml kanamycin was inoculated with a single colony and incubated overnight in a shaker incubator at 37 °C and 300 rpm to obtain a starter culture. The starter culture was added at a 1:1000 ratio to the culture media (TBp media or ZYM5052, supplemented with 50 µg/ml kanamycin) and incubated at 37 °C with vigorous shaking at 300 rpm until the OD_600_ reached 0.5–0.7. At this point, IPTG was added to the culture to a final concentration of 1.0 mM to induce expression. The induced culture was incubated at 25 °C overnight at 300 rpm. The overnight culture was centrifuged at 13,000x*g* for 2 min at 4 °C to obtain a wet cell pellet. The supernatant was collected and analysed as the extracellular fraction. The pellet was stored at − 20 °C and processed within a week to avoid sample degradation.

### Subcellular fractionation

The periplasmic fraction of cells was prepared using the protocol as described by Zhang and colleagues (2018). Briefly, the cell pellet was resuspended in ice-cold periplasmic extraction buffer I (20% w/v sucrose in 100 mM Tris HCl, pH8.0; 1 ml per 20 ml culture). The mix was incubated on ice for 30 min and pelleted at 10,000x*g* for 10 min at 4 °C. The supernatant was carefully removed using a pipette and the pellet was resuspended (1 ml per 20 ml culture) in periplasmic extraction buffer (50 mM MgCl_2_ in 100mM Tris HCl, pH 8.0). The mix was incubated on ice for 20 min and pelleted at 10,000x*g* for 10 min at 4 °C. The supernatant was recovered and combined with the previous fraction to represent the periplasmic fraction of the cells. The pellet was stored at − 20 °C during the preparation of cytoplasmic fraction.

### Preparation of cytoplasmic fraction

The cytoplasmic fraction of the cells was prepared using the protocol as described by Zhang et al. (2018). The pellet from the periplasmic protein preparation was resuspended (3 ml per 20 ml culture) in lysis buffer (50 mM Tris, 150 mM sodium chloride, pH 8.0). The suspension was sonicated on ice using 6 × 30 s bursts at a setting of 30−40% amplitude, followed by 30 s rest on ice, repeated three times. The suspension was cooled on ice for several minutes between each burst. The sonicated mix was pelleted at 12,000x*g* for 15 min. The supernatant was collected as the cytoplasmic fraction of the cells.

### Purification of AmCut using affinity chromatography

AmCut purification was performed using Protino® Glutathione Agarose following the manufacturer’s procedure. The cell lysate was centrifuged at 10,000x*g* for 30 min at 4 °C and filtered through a 0.45 μm membrane. 1.333 ml of agarose suspension (1 bed volume resin) was transferred into a column and equilibrated using phosphate-buffered saline (PBS). The cell lysate was added to the column and equilibrated at room temperature for 30 min. After washing the gel was equilibrated with PreScission Protease buffer. 10 U/ml PreScission Protease was added to the column and incubated for 16–18 h at 4 °C. The column was washed using 5 bed volumes of Elution buffer (50 mM Tris-HCl and 10 mM reduced glutathione, pH 8.0) in triplicate.

### Assay of cutinase activity

Cutinase activity was estimated spectrophotometrically as outlined by Winkler and Stuckmann (1979), with the modifications previously described (Dheeman et al. 2010), using para-nitrophenol palmitate (*p*-NPP) as the substrate. Cutinases have been shown to use para-nitrophenol esters as substrates (Kim et al. 2003). Briefly, the substrate solution was prepared as follows; Solution A was 0.34% w/v Triton X-100, 1.15 mM CaCl_2_ in 57.47 mM Tris-Cl (pH 7.5). Solution B was prepared by mixing *p-*NPP in isopropanol and acetonitrile in 4:1 ratio to reach a final concentration of 20mM. The solution was stored at − 20 °C until needed. Prior to use, Solution A was heated in a 60 °C water bath and solution B was added to solution A in the ratio of 17.4:1 to reach the final concentration of 0.3% w/v Triton X-100, 1 mM CaCl_2_ in 50 mM Tris-Cl, 1 mM *p-*NPP, 4% v/v isopropanol and 1% v/v acetonitrile.

Spectrophotometric data were captured with a Greiner CELLSTAR® 96 well plate reader, using a flat bottom plate. Each well contained 230 µl final substrate solution and 20 µl cutinase solution as appropriate. Upon mixing, the plate was incubated for 15 min at the 37 °C. The release of *p*-nitrophenol (*p*-NP) was monitored at 400 nm. All experiments were performed in triplicate. A blank, using 50mM Tris-Cl (pH 7.5) in the absence of cutinase, was included. The concentration of *p*-NP was calculated using a standard curve of 0–100 µM *p*-NP. One international unit (IU) of cutinase was defined as the amount of enzyme needed to release 1µmol of *p*-NP per minute under specific assay condition (see eg., Duan et al. 2017).

### Zymogram for cutinase detection using 4-methylumbelliferyl butyrate (MUFB)

Lipolytic activity of proteins separated by non-reducing SDS PAGE gel was detected by zymogram analysis before Coomassie staining of the gels. After electrophoresis, gels were soaked for 30 min at room temperature in 2.5% v/v Triton X-100 in 50mM phosphate buffer (pH 7.0), washed in 50 mM phosphate buffer (pH 7.0) for 20 min at room temperature, and then submerged in a solution of 100 µM MUFB in the same buffer. The gel was incubated in the substrate solution until the activity bands became visible under UV illumination (< 5 min).

### Western blot

Following electrophoresis, proteins were transferred to a polyvinylidene fluoride membrane and incubated in phosphate-buffered saline tween (PBS-T) with 10% v/v blocking buffer and HRP-conjugated anti-GST antibody (1:5000 dilution) overnight in a shaker at 4 °C. The next day the membrane was incubated with HRP substrate, *p*-coumaric acid, and visualised using luminol.

## Results

### The AmCut gene and signal peptide

**Fig. 1:**
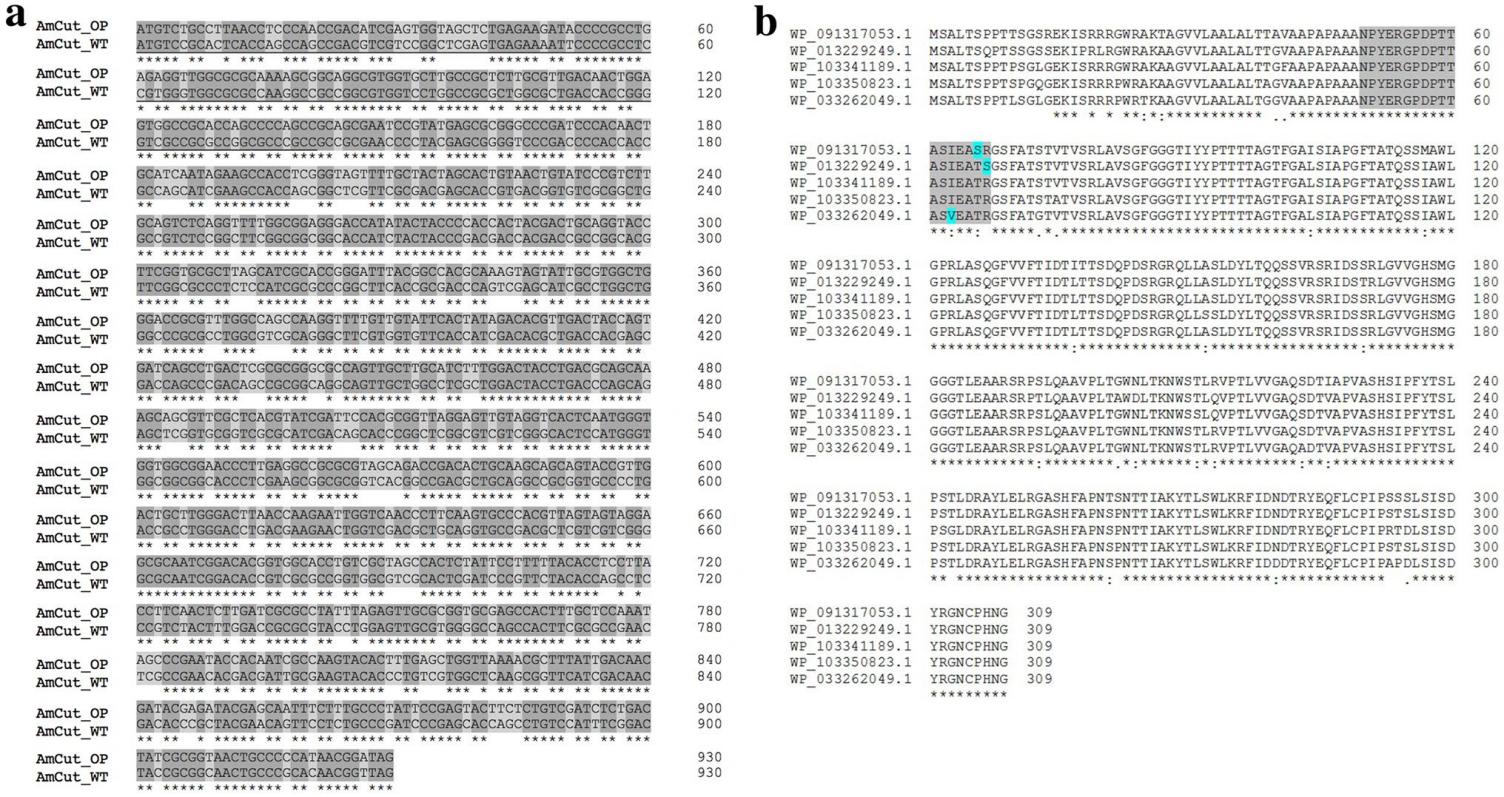
**a** The nucleotide sequence alignment of the codon optimised version of *A. mediterranei* cutinase (AmCut) gene (AmCut_OP; GeneBank Accession Number: MZ424082) with its native version (AmCut_WT; NCBI RefSeq: NC_014318.1:8278536–8279465). Identical nucleotides are highlighted in dark grey while different nucleotides are highlighted in light grey. The underlined nucleotide sequences represent the signal peptide encoding sequence. **b** Multiple sequence alignment of first six BLASTp hits for the input sequence of AmCut showing the N-terminal sequence highlighted in grey. These six BLASTp hits were: Alpha/beta hydrolase from *Amycolatopsis tolypomycina* (WP_091317053.1), lipase from *Amycolatopsis mediterranei* (WP_013229249.1), alpha/beta hydrolase from *Amycolatopsis* sp. CA-126,428 (WP_103341189.1), alpha/beta hydrolase from *Amycolatopsis* sp. CA-128,772 (WP_103350823.1), lipase from *Amycolatopsis vancoresmycina* (WP_033262049.1). The leader sequence is upstream of the greyed out N-terminus sequence used for database interrogation

The AmCut gene was known from previous studies (Tan et al. 2021). It was a 930 bp gene with GenBank ID of ADJ49206. For the studies reported herein, the AmCut gene sequence was codon optimised and synthesised *de novo* using a commercial service (Fig. 1a). Figure 1b shows a BLASTp search of the database for sequences related to AmCut. Clearly, AmCut is related to a number of similar proteins in *Amycolatopsis* species. In all cases, the N-terminus was found to be preceded by a 49 amino acid signal peptide. In the case of AmCut that sequence was: MSALTSQPTSSGSSEKIPRLRGWRAKAAGVVLAALALTTGVAAPAPAAA (see Fig. 1a,b).

**Fig. 2:**
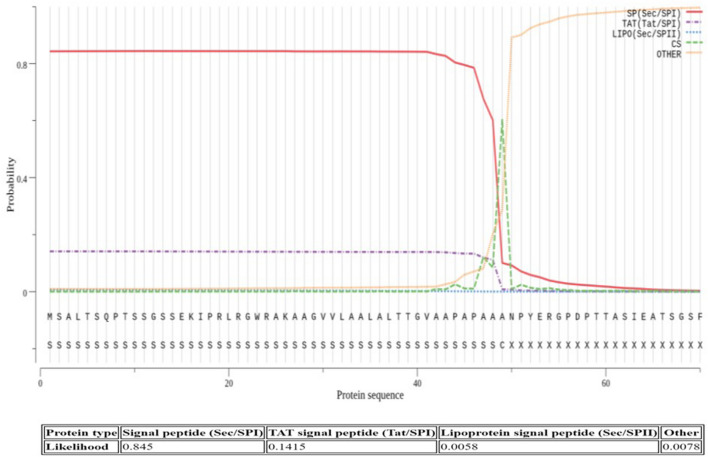
The analysis of the putative signal peptide sequence in the AmCut gene using SignalP-5.0. Sequence 1–49 was predicted to be a Sec signal peptide (Probability = 0.845) with the cleavage site most likely to be between amino acids 49 and 50. The probability that the peptide was either a TAT Signal peptide or Lipoprotein signal peptide was low at 0.1415 and 0.0058 respectively

The presence of a N-terminal leader sequence showed that AmCut was probably translated as a larger protein and subsequently processed by removal of an N-terminal peptide. For an extracellular enzyme, it was not unexpected that the N-terminus might be associated with an upstream signal peptide. Such peptides are common in secreted proteins (Freudl 2018; Schneewind and Missiakas 2012). The signal peptide is used to direct AmCut transport across the single cell wall of *Amycolatopsis* into the extracellular environment. It is estimated that for some bacteria, as many as 10% of all proteins have signal peptides (Ivankov et al. 2013). Predictive analysis using SignalP-5.0 confirmed that the upstream leader sequence was consistent with its being a signal peptide responsible for protein translocation through the *Sec* secretion pathway (SignalP-5.0 probability, 0.845; see Fig. 2). It also showed that the predicted cleavage site for the signal peptide coincided with the N-terminus of the purified protein (Fig. 1b).

Thus, we established that AmCut is likely to be secreted across the cell membrane of *A. mediterranei via* the Sec secretion pathway.

### Cloning of AmCut with signal peptide in place

**Fig. 3:**
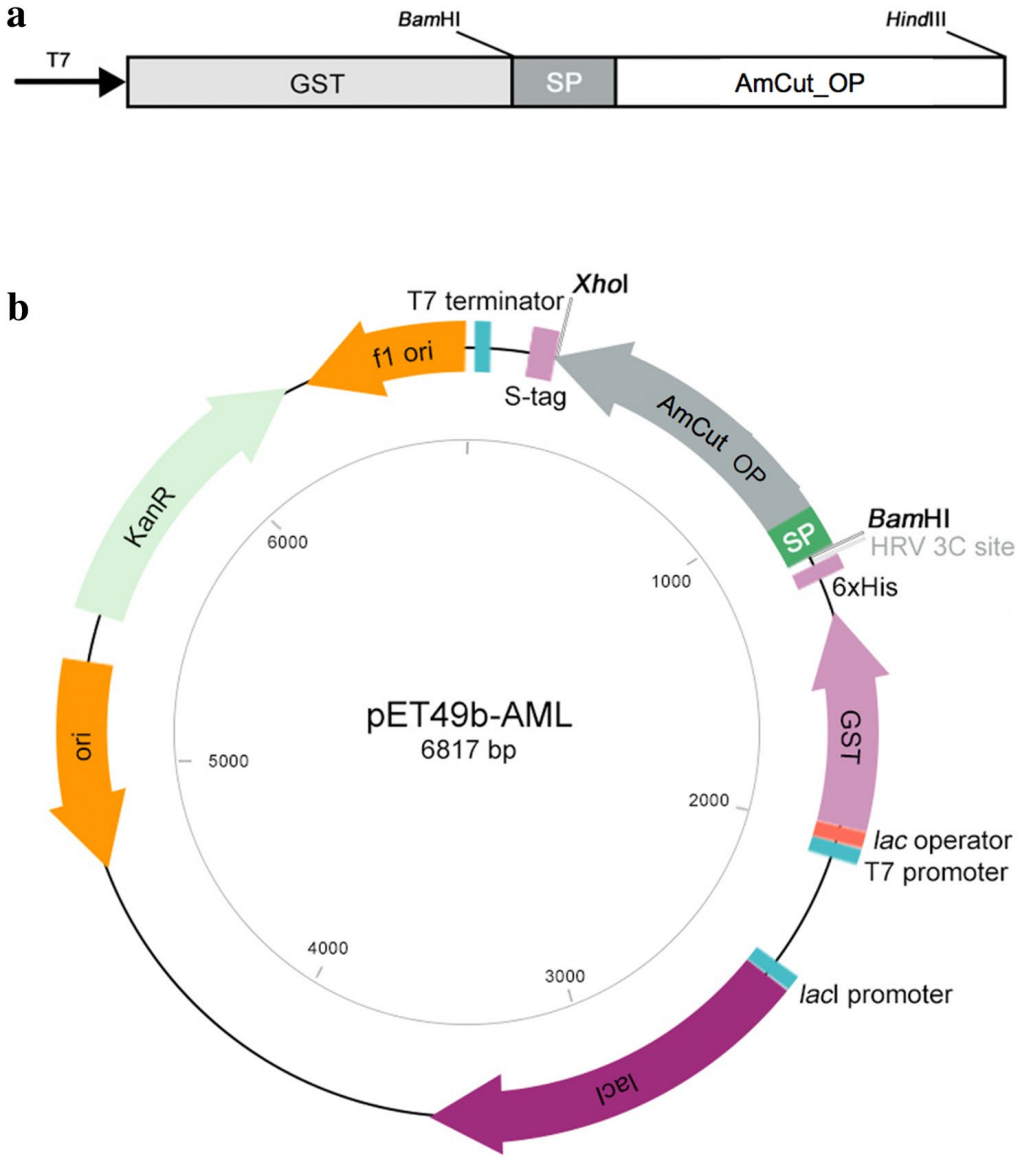
**a** A schematic of the cloned construct comprising the signal peptide (SP) located upstream of the AmCut gene (AmCut_OP) which resulted in an extracellularly secreted protein following expression. **b** The construct also contained a GST tag present in the pET49b vector to facilitate affinity purification. The signal peptide lies between the GST tag and the AmCut gene separated by a linker region. Following expression, the GST tag was removed using a specific protease

Previous studies in this laboratory had cloned a truncated AmCut gene lacking the N-terminal signal peptide and showed that the enzyme was expressed as a soluble protein in *E. coli* cytosol (Tan et al. 2021). To further examine the role of the signal peptide on AmCut activity, the entire AmCut gene, *including* the putative signal peptide, was cloned (Fig. 3). It was of interest to examine the effect, if any, of the predicted leader sequence on activity and expression in *E. coli*. Cloning employed a GST tag, which is known to enhance recombinant protein solubility (Rosano and Ceccarelli 2014).

**Fig. 4:**
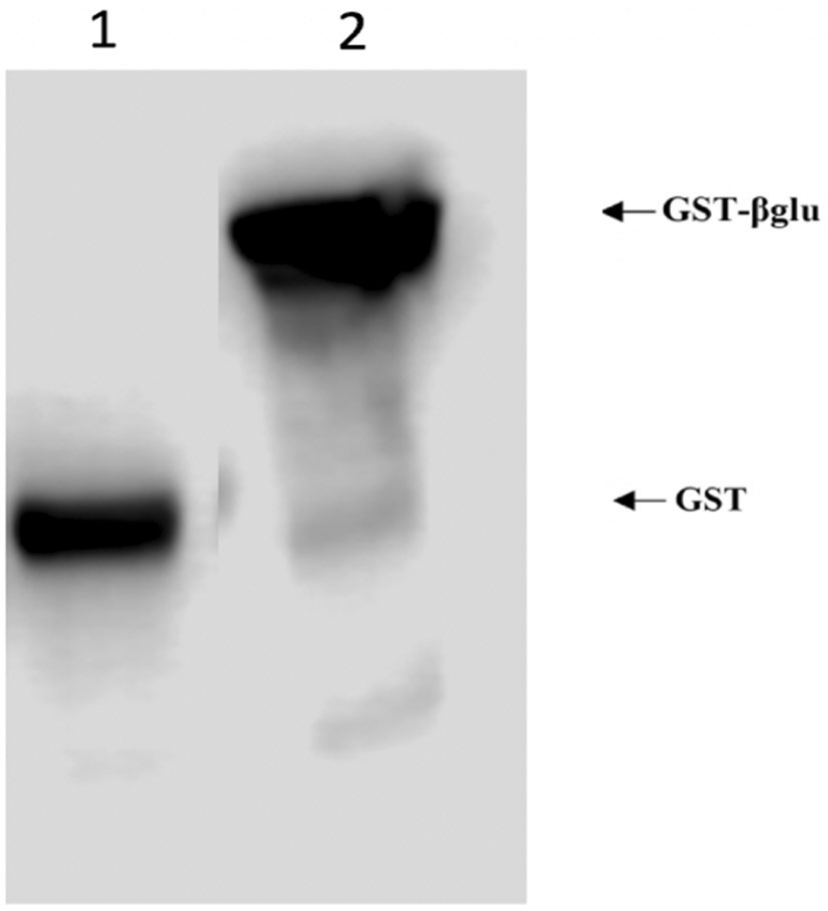
Western blotting of IPTG-induced pET49b-AmCut expression lysate using HRP conjugated anti-GST antibodies. Lane 1—pET49b-GST-AmCut expressed cell lysate; Lane 2—GST tagged β-glucosidase was used as a size guide (GST-β-glu; ~ 62 kDa). The GST-AmCut fusion protein was expected to have a size of approximately 60 kDa, while GST alone is approximately 26.9 kDa. For clarity of display redundant lanes have been removed from the gel image. For the full unedited gel see supplementary Fig. S1

The expression of this heterologous AmCut in *E. coli* BL21(DE) cells showed expression of soluble AmCut activity. For some proteins, a signal peptide can result in inclusion body formation during recombinant expression (Pulido et al. 2020; Singh et al. 2013); however, in this case, soluble AmCut was found by activity assay in cell lysate fractions. It was assumed that the enzyme was being expressed in *E. coli* cytosol with the GST tag attached (the tag was for ease of purification). However, when purification of AmCut using immobilised glutathione was attempted, no binding of the GST tagged protein to the immobilised glutathione support was observed. This was puzzling since the enzyme was clearly being expressed as shown by the activity assay. A Western blot indicated that the GST tag was also expressed; however, it was without the attached AmCut protein as judged by its size relative to a control (see Fig. 4).

**Fig. 5:**
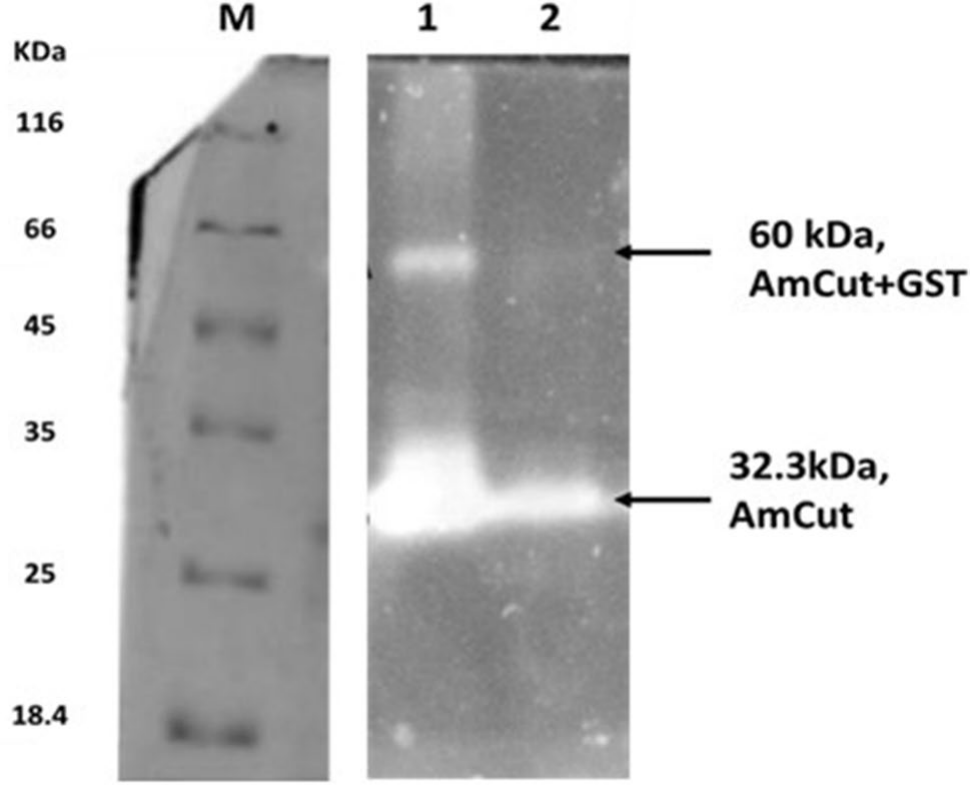
Coomassie staining (left) and 4-MUF butyrate zymogram (right) analysis of cell lysate. Lane M- molecular weight markers. Lane 1—Zymogram of GST-AmCut cell lysate expressing BL21(DE3); Band A and band B are estimated as approximately 60 kDa and approximately 26 kDa respectively using relative migration distance. Lane 2 shows a *Mucor meihei* lipase (Sigma Aldrich; ~ 32 kDa) as a positive control and size marker. For clarity of display redundant lanes have been removed from the image. For the full zymogram see supplementary Fig. S2

Figure 4 shows the expression of the GST protein at a molecular weight considerably lower than the GST-tagged *Mucor meihei* glucosidase used as a marker. If the AmCut protein was attached to the GST tag the combined molecular weight was expected to be approximately 60 kDa. Clearly, the GST tag is being expressed, but without the AmCut attached. This seemed to indicate that the GST tag was being cleaved from the GST-AmCut construct, after its expression, by an *E. coli* peptidase. This hypothesis was further confirmed using zymogram analysis. A non-reducing SDS gel of a cell lysate was run and a portion of the gel with marker proteins was excised and stained for protein using Coomassie blue. The rest of the gel was subjected to zymogram analysis and the presence of cutinase activity was detected using a MUF-butyrate overlay assay (Fig. 5).

Figure 5 shows that the majority of cutinase activity is at approximately 30 kDa (the expected molecular weight of AmCut alone) while a much smaller portion is present at approximately 60 kDa (AmCut with attached GST). These results indicated that while GST and AmCut were both being expressed in *E. coli* BL21(D3), the GST tag and the AmCut protein had become separated. It was clear that this could only be explained if the AmCut signal peptide was being recognised by *E. coli* BL21(D3) peptidases and cleaved, thereby separating the GST tag and the AmCut protein (Jeong et al. 2009). To confirm this hypothesis, it was necessary to examine the AmCut gene without the signal peptide.

### Expression of AmCut without the signal peptide

**Fig. 6:**
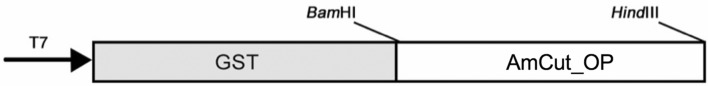
The expression construct following the cloning of AmCut without the signal peptide

The truncated AmCut gene was expressed, as outlined previously (Tan et al. 2021), without the signal peptide (see Fig. 6). The GST tag was still retained to facilitate purification. In this case, soluble expression was again observed, but purification using the immobilised glutathione was now possible. This confirmed that the signal peptide was responsible for the separation of the GST tag and the AmCut protein.

### Purification of AmCut without signal peptide

**Fig. 7:**
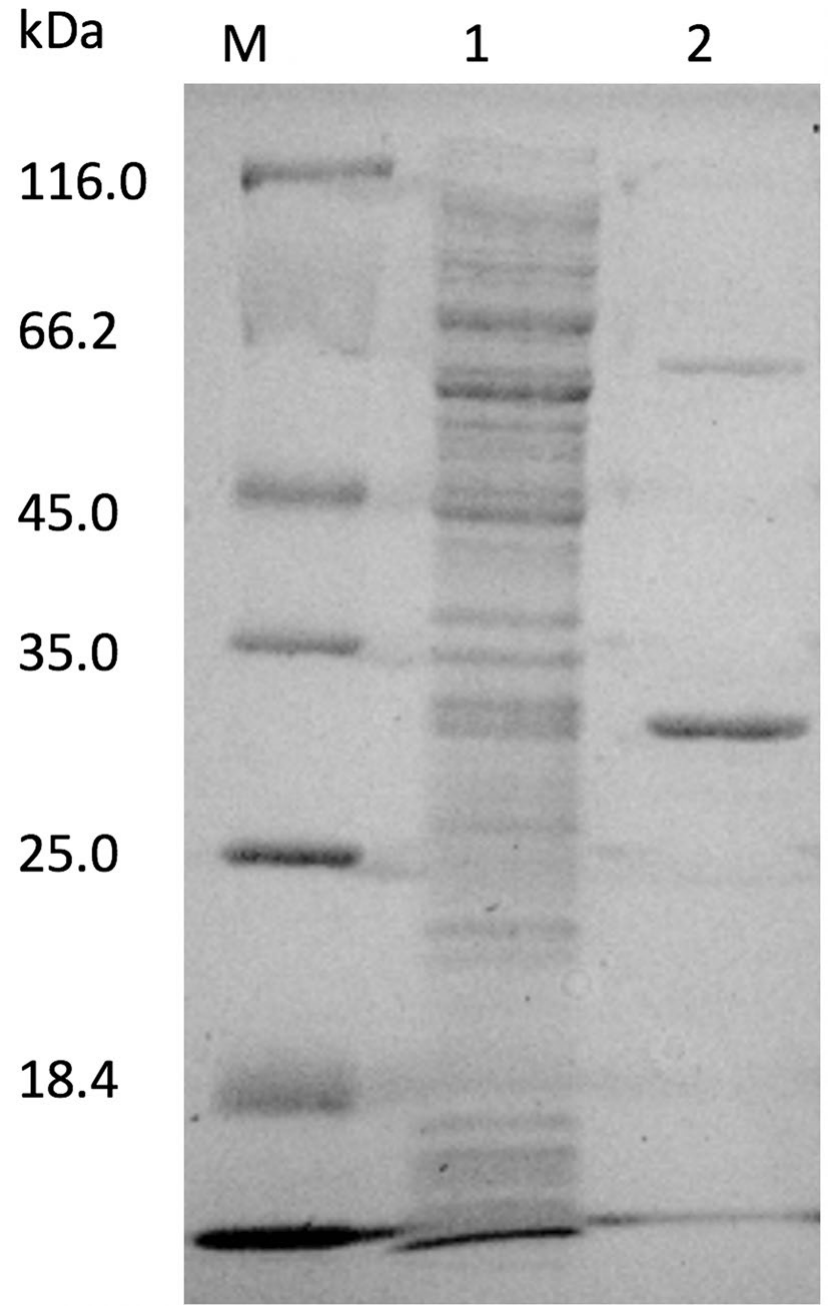
Coomassie blue staining analysis of SDS electrophoresis gel of crude lysate of AmCut and the purified AmCut using Glutathione Sepharose 4B resin. The AmCut was liberated by cleavage using PreScission Protease. M – Pierce Unstained Protein MW Marker, 1—Crude lysate of AmCut, 2—Purified and GST-cleaved AmCut

The recombinant AmCut readily bound to immobilised glutathione following the removal of the signal peptide. Figure 7 shows a 12% v/v SDS gel of the purified enzyme as eluted from a glutathione affinity column following *in situ* cleavage of the GST tag using PreScission Protease.

Figure 7 shows that a small portion of the recombinant AmCut was not fully cleaved (minor band at 60 kDa approximately) by the PreScission Protease which is commonly observed with GST tagged enzymes. Nonetheless, it is clear that the purification was successful, and that the GST tag bound AmCut to the column. A yield of approximately 20% was obtained for the affinity purification step (Table 1).

**Table 1:** Purification table of AmCut through affinity chromatography using Glutathione Agarose 4B as the resin and PreScission Protease for on-column GST tag cleavage

	Total recovery		Specific activity (U/mg)	Fold purified	% Yield
	Protein (mg)	Enzyme (U)			
Initial starting material	24.5	151.8	6.2	--	--
Glutathione affinity chromatography	0.17	30.2	173.6	28.0	19.9

### Subcellular distribution studies

**Fig. 8:**
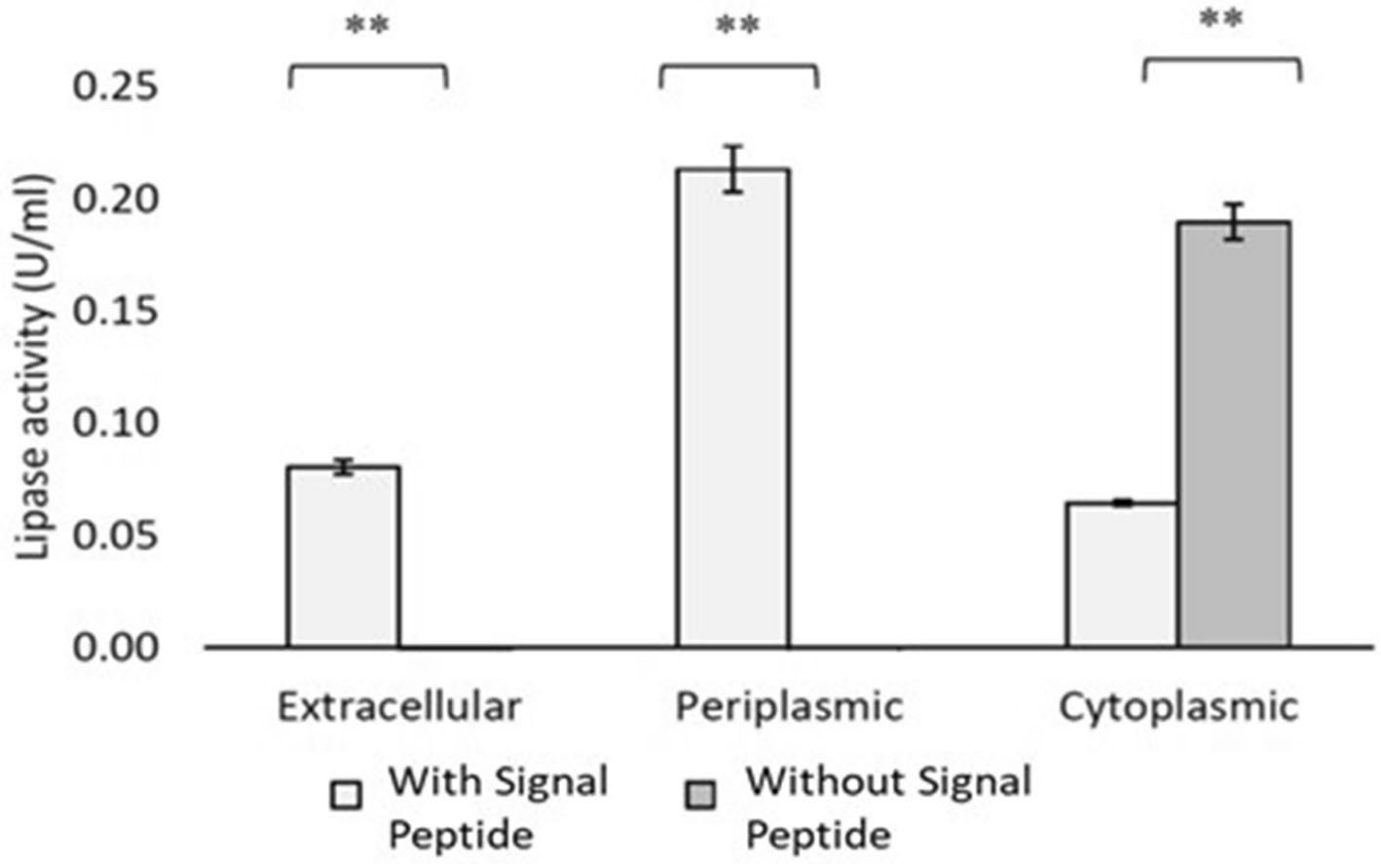
The distribution of active cutinase from pET49b-AmCut construct (AmCut with signal peptide attached) and pET49b-AmCut_SP construct (AmCut without signal peptide) as expressed in extracellular, periplasmic, and cytoplasmic fraction of BL21(DE3) induced with 1.0 mM IPTG at OD_600_ = 0.6–1.0 (25 °C, 20 h). All experiments were carried out in triplicate and the differences between the mean of each fraction were analysed using one-tailed t-test (paired two samples for mean). Samples with a statistically significant difference were marked with asterisks (**p < 0.05)

To further explore the expression of the AmCut protein in the presence or absence of the signal peptide *E. coli* BL21(DE) cell fractions were prepared. This involved preparation of an extracellular fraction, a cytosolic fraction and a fraction containing proteins of the periplasmic space. Figure 8 shows a comparison of the distribution of AmCut activity in subcellular fractions with and without the signal peptide.

This experiment led to an interesting observation; when AmCut was expressed in *E.coli* BL21(DE3) cells in the presence of the signal peptide, it was mainly found in the periplasmic space and extracellular medium. However, when the signal peptide was omitted, expression was exclusively in the cytosol of *E. coli*. Thus, the signal peptide, native to the Gram-positive *Amycolatopsis*, was recognised in the Gram-negative *E.coli* and directed expression to the periplasmic space. This occurred even when the GST tag was present upstream of the signal peptide. It seems that the presence of the GST tag neither hindered the cleavage of the signal peptide nor the transport of the AmCut protein to the periplasm via the Sec secretion pathway. Figure 8 shows that when the signal peptide was present there was detectable activity in the periplasm *and* in the extracellular space. Thus, we conclude, that cutinase that is directed to the periplasm is also capable of passing through the *outer* membrane. Given its activity as an esterase, it was possible that it was partially hydrolysing the outer membrane thereby facilitating its own release. Previous studies have noted the ability of hydrolase enzymes to render the *E. coli* outer membrane permeable and this property was exploited by some workers as a method to enhance the secretion of recombinant proteins into the culture medium (Zhou et al. 2018; Su et al. 2013). However, arguing against this, is the observation that the enzyme without the signal peptide did not migrate out of the cytosol. This appears to argue for a non-classical secretion pathway for outer membrane translocation (Wang et al. 2016). This combination of Sec pathway for inner membrane translocation followed by non-classical transport across the outer membrane is unusual.

## Discussion

In this study, the *Amycolatopsis mediterranei* cutinase (AmCut) gene showed a high degree of similarity to a number of related enzymes. Most were lipases, or putative esterases, from *Amycolatopsis* and related species. A signal peptide sequence was identified for these enzymes. Predictive software analysis of the AmCut signal peptide sequence provided clear evidence that it was similar to those associated with transmembrane transport via the Sec pathway. This pathway is known to be highly conserved across species (Kang and Zhang 2020). This was further supported by the AmCut migration into the periplasmic space of *E. coli* observed in this study.

Since the signal peptide associated with the native *A. mediterranei* AmCut enzyme was capable of transporting AmCut into the periplasmic space of *E. coli* BL21(DE); it may also be a useful sequence for targeting of other proteins to this space. While it is not unknown for signal peptides from other organisms to function in *E. coli* due to the high degree of conservation of such transport machinery (see Jalal et al. 2011), it is seldom reported. It was also unusual to find one that is functional in the presence of a bulky upstream GST tag. This is not always the case, and signal peptides associated with certain lipases have not proven to be functional in *E. coli* (see Pulido et al. 2020). As this study shows, the signal peptide may be placed between bulky proteins (as demonstrated here with GST and AmCut) and still function for secretion in *E. coli*. This may be of interest for fusion proteins that require subsequent processing to liberate individual proteins or peptides, such as in the recovery of enzymes as part of a bioprocess (Zhou et al. 2018).

In the presence of the signal peptide, the expressed enzyme was found in the periplasmic space. Previous studies have shown that the expression of cutinases in this space can lead to membrane permeabilisation and can enhance the extracellular export of co-expressed target proteins (Su et al. 2013) in a process known as periplasmic leakage (see Zhou et al. 2018 for review). The recombinant *Amycolatopsis mediterranei* AmCut, and associated optimised expression vector, examined in this study may be a useful biotechnological tool for such applications. In the absence of the signal peptide expression was only in the cytosol and no activity was observed in other fractions.

The presence of a confounding native *E. coli* lipase activity in BL21 cells of 18-20 kDa was recently reported (Buruaga-Ramiro et al. 2020). It was possible that this enzyme might be interfering with the interpretation of the AmCut findings in this study. However, this was considered unlikely for a number of reasons: firstly, the subcellular distribution data of Fig. 8 showed no activity in periplasmic and extracellular cell fractions prepared from expression of the AmCut gene *without* its signal peptide. Therefore, we can conclude that there are no interfering activities in the periplasm or external medium. Of course, it is still possible that lipase activity in the cytosol could be masked by the recombinant cutinase. Even if that were the case, it does not affect the observation that the signal peptide alters the pattern of expression. Secondly, the MUF-butyrate activity stain did not show a contamination band at ca. 20 kDa. This is sufficiently different in molecular weight to be clearly separated from the AmCut protein. Finally, the enzyme purified from the *E. coli* cytosol bound to the immobilised glutathione column and was eluted as a substantially pure protein of the correct molecular weight (Fig. 7) which is not possible for a contaminating lipase.

*T. fusca* cutinase is, perhaps, the most prominent previous example of an *E. coli* expressing a cutinase (Su et al. 2013, 2017) prior to this study. Unlike *T. fusca* lipase A, the expression of AmCut without signal peptide in the cytosol of *E. coli* neither led to its secretion into the medium, nor did it lead to production of inclusion bodies. Thus, the ability of AmCut to digest *E. coli* membranes seems to be much less than that of the *T. fusca* cutinase. On the other hand, when the signal peptide was present, AmCut was transported to the periplasmic space, and from there to the extracellular culture medium. The expressed *T. fusca* cutinase differed from the AmCut construct of this study in three aspects. Firstly, the *T. fusca* enzyme did not require a signal peptide for secretion into the culture medium. By contrast, AmCut was *only* observed in the culture medium when expressed with its signal peptide. Secondly, the *T. fusca* cutinase was translocated though the *E. coli* inner membrane to the periplasmic space by digestion of phospholipids in the inner membrane. This was proven when a mutated inactive *T. fusca* enzyme was unable to cross the inner membrane of *E. coli* and was not secreted into the culture medium. By contrast, active AmCut expressed in the cytosol (without signal peptide) was retained in this compartment. This suggests that its ability to degrade *E. coli* membranes is much less for AmCut than for the *T. fusca* cutinase. Thirdly, the *T. fusca* enzyme was able to digest the outer membrane of *E. coli* and enter the culture medium. For AmCut, expression in the culture medium was also observed, but is unlikely to be due to outer membrane digestion. Rather, it is probably entering the culture medium, either by passive leakage, or by non-classical translocation routes (Wang et al. 2016).

In terms of future biotechnological application of AmCut, it has recently been shown to readily degrade commercial ester-based polymeric substrates (Tan et al. 2021). Therefore, the extracellular expression of AmCut described in this present study offers significant opportunities for sustainable biocatalytic degradation of common polyester-based plastics.

Modelling studies of AmCut’s 3-D structure indicated possible disulphide bonds stabilising its structure (Tan et al. 2021). Disulphide bonds will not be formed in *E. coli’s* cytosol but will be formed in the periplasmic space (see e.g. Ke and Berkmen 2014). This is a significant advantage of periplasmic expression. The formation of disulphides clearly does not affect AmCut activity (from this study) but could potentially increase its stability. Moreover, periplasmic expression offers the possibility to introduce additional stabilising disulphides by mutagenesis. The ease of gene manipulation in *E. coli* will allow for introduction or removal of disulphides as appropriate for specific applications.

Overall, we have shown that in the presence of the signal peptide we can produce a modified *E. coli* that is able to secrete this robust, solvent stable extracellular cutinase into the culture medium.

In conclusion, this study analysed a recently reported sequence from *Amycolatopsis mediterranei* that coded for a cutinase (AmCut) accompanied by a novel N-terminal leader signal. AmCut, with its signal peptide, is unique in being expressed as an extracellular enzyme in both gram-positive and gram-negative cells. Cloning and subsequent over-expression in *E. coli*, coupled with subcellular fractionation studies, provided clear evidence that the signal sequence was associated with a functional transmembrane transport process via the Sec pathway. Utilising the recombinant expression system described herein, the production of functional recombinant cutinase can be targeted to the *E. coli*, periplasm and extracellular culture medium. Such a tuneable organism will be biotechnologically useful in applications where breakdown of lipids or other polyesters in the environment is required. Further studies in this area are ongoing.

## Electronic Supplementary Material

Below is the link to the electronic supplementary material.

Supplementary file1 (DOCX 341 KB)

## Data Availability

Data and materials are available upon request.
