# Supplementary material for: Extracellular secretion of a cutinase with polyester-degrading potential by E. coli using a novel signal peptide from Amycolatopsis mediterranei
Source: World J Microbiol Biotechnol. 2022 Feb 23;38(4):60. doi: 10.1007/s11274-022-03246-z (PMC8866283; doi:10.1007/s11274-022-03246-z)
Supplement: Supplementary file 1 — Supplementary file1 (DOCX 341 KB) [file 11274_2022_3246_MOESM1_ESM.docx]

Extracellular secretion of a cutinase with polyester-degrading potential by *E.coli* using a novel signal peptide from *Amycolatopsis mediterranei*

*Yeqi Tan* ^a^*, Gary T. Henehan* ^a^*, Gemma K. Kinsella* ^a^*, Barry J. Ryan* ^a, *^

^a^ School of Food Science and Environmental Health, Technological University Dublin, Grangegorman, Dublin 7, D07 H6K8, Ireland

^*^ [barry.ryan@TUDublin.ie](mailto:barry.ryan@TUDublin.ie); Ph: 00353-1- 220 5671

ORCID: 0000-0001-7213-3273

**Supplementary Material:**

Figure S1 is an expanded version of Figure 4 of the main manuscript. This experiment was originally designed to show the effect of glucose levels on suppression of GST-tagged AmCut expression (catabolite repression) to prove it was a recombinant enzyme. It was expected that the expression of the GST-AmCut construct would decrease as glucose bound to the *lac* operon. A size marker (GST-tagged *Mucor meihei* glucosidase; ~62kDa, Lane 9) was included as a control. The GST-AmCut recombinant protein should run at roughly the same molecular weight as the GST-glucosidase construct (Lane 9). However, the GST tag appeared at a much lower molecular weight than expected indicating that it had been cleaved from AmCut. Increasing glucose in the medium supressed the GST tag, as expected: 1% glucose in the medium clearly reduced expression (Track 7). This experiment was the first evidence that *E. coli* peptidases were cleaving the GST tag from the GST-AmCut construct after its expression. Figure 4 of the main manuscript used tracks 2 and 9 of this gel.


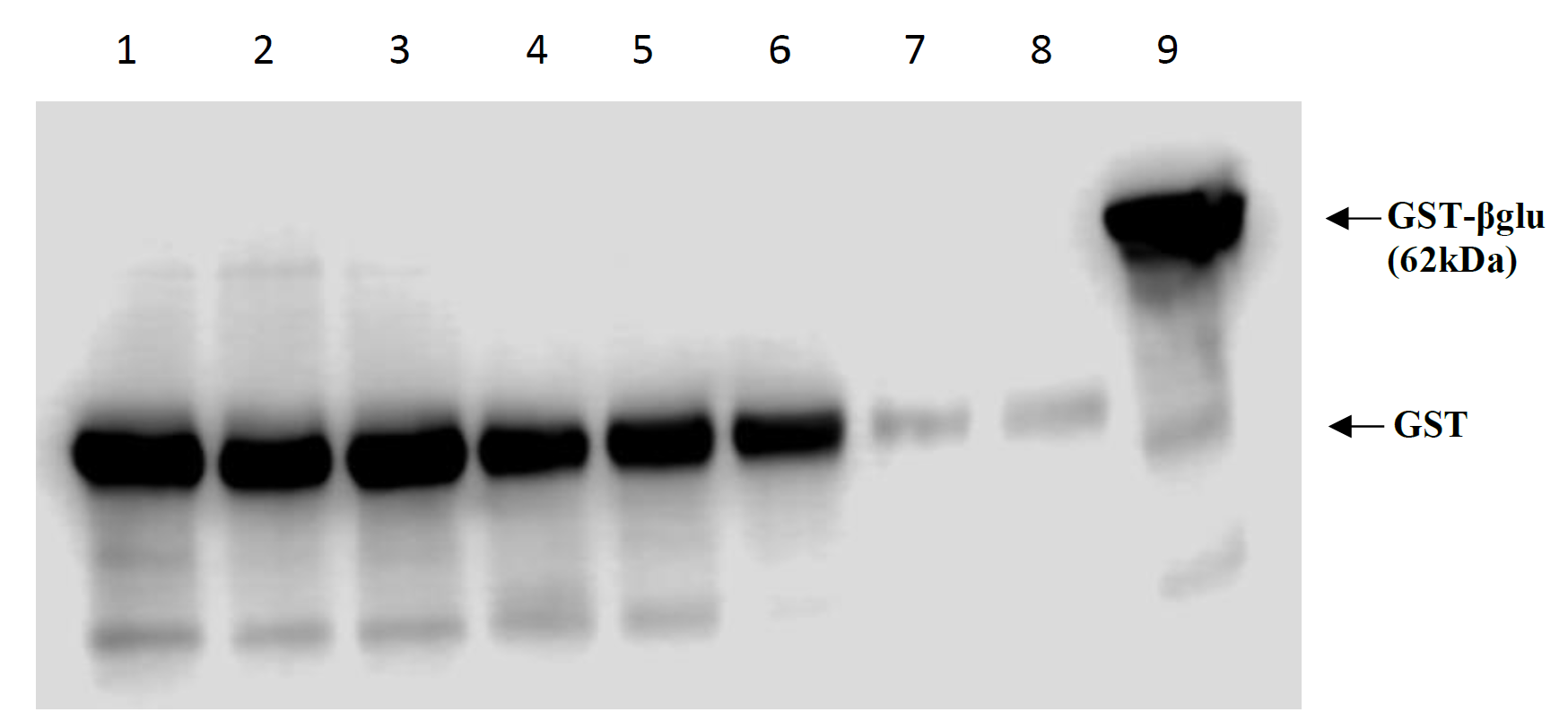


**Figure S1:‑** Western blotting of IPTG-induced pET49b-AML expression at 25^o^C overnight at different glucose concentrations using HRP conjugated anti-GST antibodies. Lanes 1,2 – pET49b-AmCut (no glucose control); 3 – 0.05% (w/v) glucose; 4 – 0.1% (w/v) glucose; 5 – 0.25% (w/v) glucose; 6 – 0.5% (w/v) glucose; 7 – 1% (w/v) glucose; 8 –2% (w/v) glucose; 9 – GST tagged *Mucor meihei* β-glucosidase as positive control and size guide (*c.a.* 62kDa).

Figure S2 is an expanded version of Figure 5 of the main manuscript. The figure shows the zymogram staining of cell lysates expressing GST-tagged AmCut.


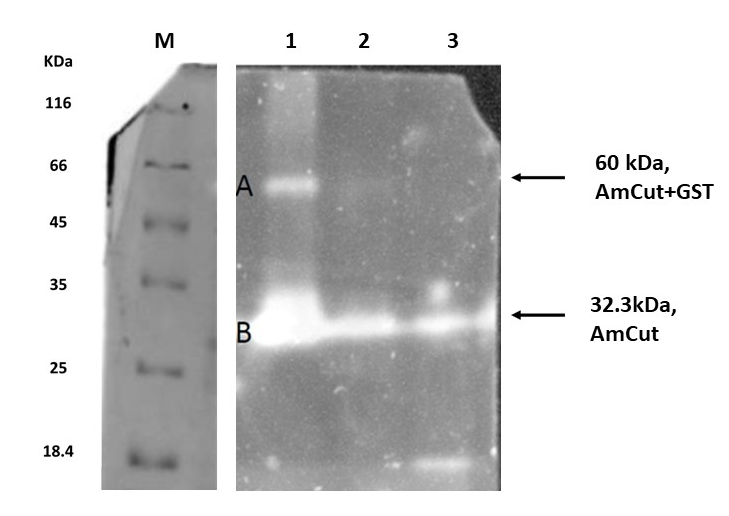


**Figure S2:** Coomassie staining (left) and 4-MUF butyrate zymogram (right) analysis of cell lysates. Lane M- molecular weight markers. Lane 1 - GST-AmCut expressed in BL21(DE3); Band A and band B are estimated as approximately 64.7kDa and 28.4kDa respectively using relative migration distance. Lane 2 shows a *Mucor meihe*i lipase (Sigma Aldrich; ~32kDa) as a positive control and size marker. Lane 3 shows AmCut expressed without the - GST tag in BL21(DE3).

This gel shows that activity staining of the GST-AmCut expressing cells (Lane 1) clearly has two bands of lipase activity in cell lysates. An upper band corresponding to the AmCut with the GST tag attached and a lower band without the GST tag. This gel is further evidence that the GST tag and the AmCut enzyme have been split apart by *E. coli* peptidases.
